# Histological transformation from non-small cell to small cell lung carcinoma

**DOI:** 10.1136/bcr-2016-216232

**Published:** 2016-08-19

**Authors:** Yuki Haruta(Inoue), Yuki Kataoka, Junichi Nikaido, Naoki Nakajima

**Affiliations:** 1 Chubu Rosai Byoin, Nagoya, Japan; 2 Department of Respiratory Medicine, Hyogo Prefectural Amagasaki General Medical Center, Amagasaki, Hyogo, Japan; 3 Department of Diagnostic Pathology, Hyogo Prefectural Amagasaki General Medical Center, Amagasaki, Hyogo, Japan

## Description

**Figure 1:**
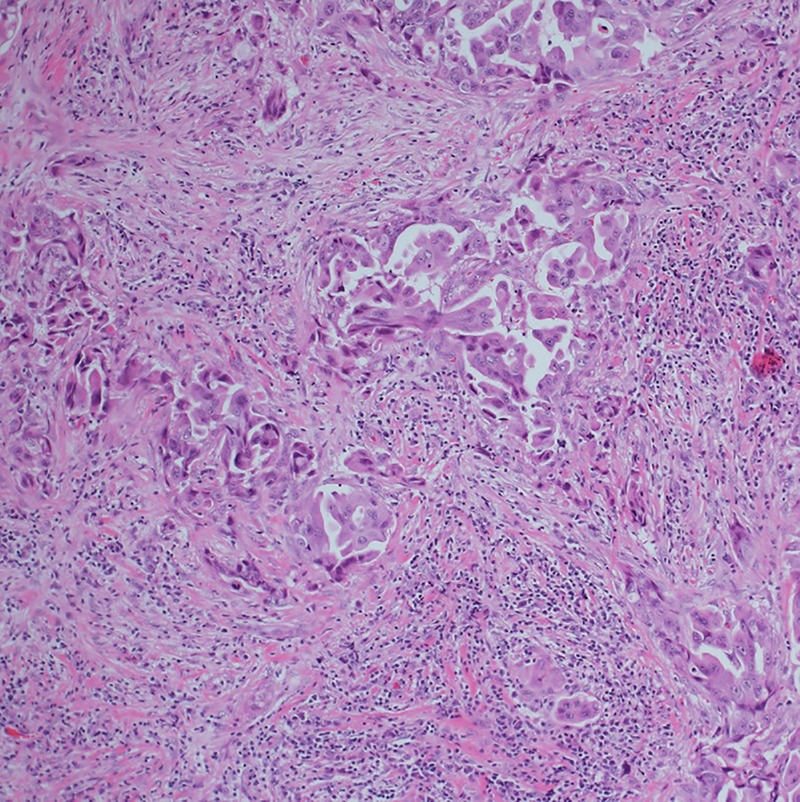
H&E staining.

**Figure 2:**
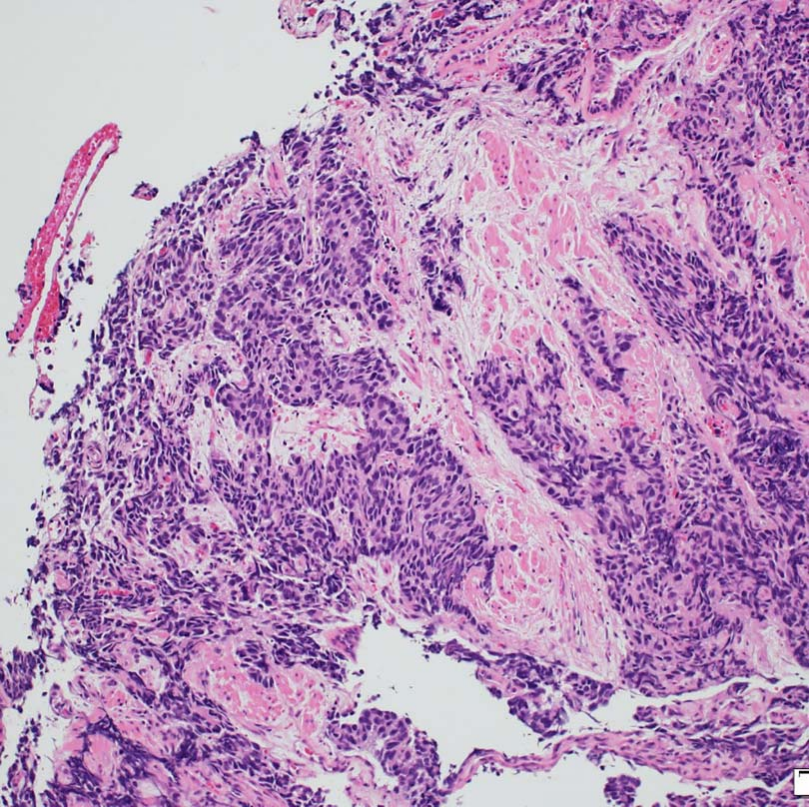
H&E staining.

**Figure 3:**
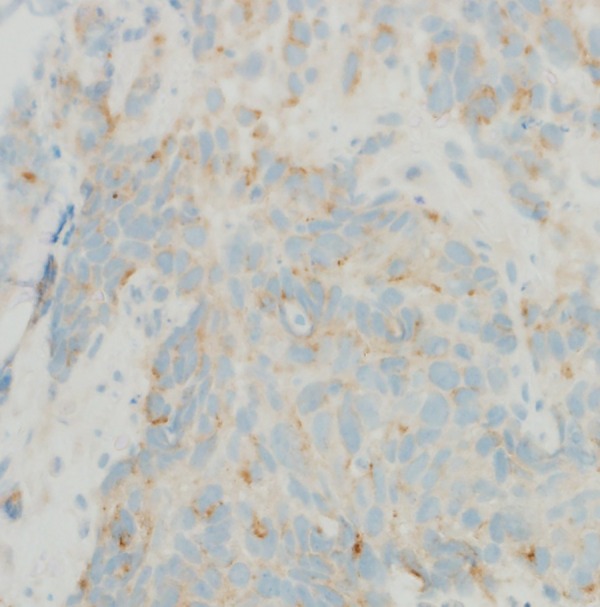
Chromogranin A staining.

**Figure 4:**
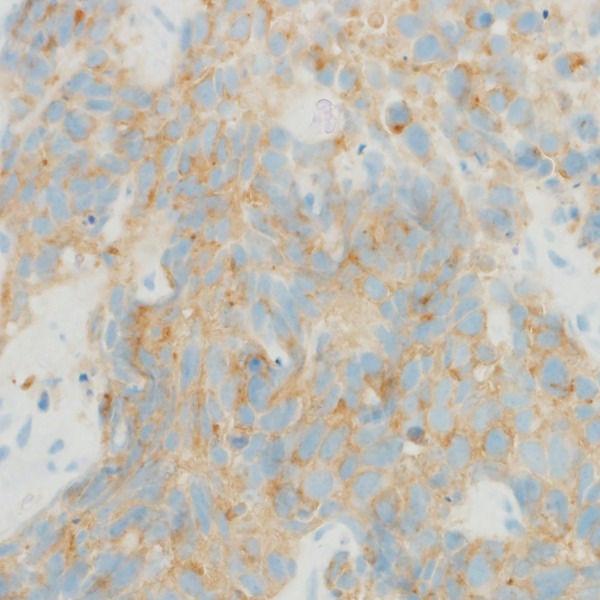
Synaptophysin staining.

A 58-year-old woman with no history of smoking was admitted to our hospital with exacerbation of cough. CT of the chest revealed a left upper lobe lung tumour. Bronchoscopic biopsy of the tumour revealed stage IV poorly differentiated adenocarcinoma (figure 1) with epidermal growth factor receptor (EGFR) gene mutation (L858R point mutation). After cytotoxic chemotherapy (cisplatin and pemetrexed) as first-line therapy, disease progression was identified. For the next 3 years, she was treated with erlotinib, an EGFR tyrosine kinase inhibitor (TKI). Regimens were changed several times (cisplatin, gemcitabine, docetaxel and afatinib) due to disease progression or adverse effects. After 2 months with afatinib, disease progression was again noted. We once again performed bronchoscopic biopsy of the primary tumour, and small cell lung cancer (SCLC) was confirmed from histopathological examination (figures 2–4). Levels of tumour markers such as progastrin-releasing peptide and non-specific elastase were elevated. Moreover, a second examination again detected the EGFR gene mutation (L858R point mutation without T790M point mutation). Amrubicin was administered, resulting in radiologically stable disease. Most cases of acquired resistance to EGFR-TKI arise from the emergence of T790M mutation, and morphological transformation to SCLC is rare.^1^ After the failure of EGFR-TKI, rebiopsy of the tumour is warranted to determine the next treatment strategy.

**Learning points**

- After the failure of epidermal growth factor receptor-tyrosine kinase inhibitor (EGFR-TKI), rebiopsy of the tumour should be performed to determine the next treatment strategy.
- But it is unclear when to perform rebiopsy after EGFR-TKI failure.

## References

[1] Cortot AB, Jänne PA Molecular mechanisms of resistance in epidermal growth factor receptor-mutant lung adenocarcinomas. Eur Respir Rev 2014;23:356–66. doi:10.1183/09059180.00004614 25176972 10.1183/09059180.00004614 PMC9487318

